# A ubiquitous and diverse methanogenic community drives microbial methane cycling in eutrophic coastal sediments

**DOI:** 10.1093/femsec/fiaf075

**Published:** 2025-07-12

**Authors:** Anna J Wallenius, Jessica Venetz, Olga M Zygadlowska, Wytze K Lenstra, Niels A G M van Helmond, Paula Dalcin Martins, Caroline P Slomp, Mike S M Jetten

**Affiliations:** Department of Microbiology, Radboud Institute for Biological and Environmental Sciences, Radboud University, 6525 AJ Nijmegen, The Netherlands; Department of Microbiology, Radboud Institute for Biological and Environmental Sciences, Radboud University, 6525 AJ Nijmegen, The Netherlands; Department of Microbiology, Radboud Institute for Biological and Environmental Sciences, Radboud University, 6525 AJ Nijmegen, The Netherlands; Department of Earth Sciences – Geochemistry, Utrecht University, 3508 TA Utrecht, The Netherlands; Department of Microbiology, Radboud Institute for Biological and Environmental Sciences, Radboud University, 6525 AJ Nijmegen, The Netherlands; Department of Earth Sciences – Geochemistry, Utrecht University, 3508 TA Utrecht, The Netherlands; Department of Microbiology, Radboud Institute for Biological and Environmental Sciences, Radboud University, 6525 AJ Nijmegen, The Netherlands; Department of Earth Sciences – Geochemistry, Utrecht University, 3508 TA Utrecht, The Netherlands; Department of Microbiology, Radboud Institute for Biological and Environmental Sciences, Radboud University, 6525 AJ Nijmegen, The Netherlands; Institute for Biodiversity and Ecosystem Dynamics, University of Amsterdam, 1098 XH Amsterdam, The Netherlands; Department of Microbiology, Radboud Institute for Biological and Environmental Sciences, Radboud University, 6525 AJ Nijmegen, The Netherlands; Department of Earth Sciences – Geochemistry, Utrecht University, 3508 TA Utrecht, The Netherlands; Department of Microbiology, Radboud Institute for Biological and Environmental Sciences, Radboud University, 6525 AJ Nijmegen, The Netherlands

**Keywords:** ANME, coastal sediment, eutrophication, methanogens, microbial methane cycle

## Abstract

Coastal areas contribute over 75% of global marine methane emissions, a proportion predicted to increase with anthropogenic eutrophication and deoxygenation. Prolonged low oxygen and high organic matter input can disrupt the methane cycle, favoring methane production over oxidation. However, factors influencing this imbalance remain unclear. Here, we show that methanogenesis dominates microbial methane cycling in the anoxic sediments of eutrophic coastal marine Lake Grevelingen (The Netherlands) after summer stratification. A shallow sulfate–methane transition zone (SMTZ; 5–15 cm depth) was observed, with high methane concentrations below. Methane was produced in all investigated layers, while methane oxidation was restricted to the narrow SMTZ. Amplicon sequencing, metagenomics, and incubations revealed a metabolically and phylogenetically diverse methanogenic community with niche separation, and methylotrophic methanogenesis prevalent in all layers. Two clades of ANME archaea, ANME-2a/b and ANME-3, were restricted to a narrow zone together with their putative syntrophic sulfate-reducing bacteria, coinciding with the observed methane oxidation activity. Our results suggest that eutrophication and deoxygenation will further contribute to rising methane emissions, tilting the microbial methane cycle toward increased methanogenesis, and decreasing the efficiency of the microbial methane filter.

## Teaser

Eutrophication favors microbial methane production over methane removal in seasonally anoxic marine basin.

## Introduction

Methanogenic archaea in marine sediments produce methane (Reeburgh 2007, Saunois et al. 2020), a greenhouse gas 86 times more potent than CO_2_ on a 20-year time scale. Atmospheric methane concentrations are rising sharply, and aquatic ecosystems, both natural and anthropogenically affected, have been estimated to be sources of at least half of global emissions (Saunois et al. 2020, Rosentreter et al. 2021). Most of the methane produced in deep marine sediments does not reach the atmosphere, as anaerobic methanotrophic archaea (ANME), in consortium with sulfate-reducing bacteria (SRB), are estimated to consume over 90% of the biologically produced methane (Knittel and Boetius 2009). This so-called methane filter, driven by sulfate-dependent anaerobic oxidation of methane (S-AOM) takes place in the sulfate–methane transition zone (SMTZ) located above the methanogenic sediment layer.

In coastal and shelf areas, the SMTZ is often found to be narrower and at a shallower depth than in the deeper parts of the oceans (Egger et al. 2018), which might affect its methane removal capacity, thus allowing methane to bypass the SMTZ and to escape to the atmosphere (Żygadłowska et al. 2024a). The drivers of the imbalance between methane production and oxidation in coastal systems are not well known, but anthropogenic activity-driven eutrophication and resulting high organic matter (OM) input and increased hypoxia often favor methanogenesis over methanotrophy (Wallenius et al. 2021).

In eutrophic systems, high sedimentation rates can lead to a shoaling of the SMTZ, and consequently, bring the methanogenic zone closer to the sediment–water interface (Egger et al. 2016, Żygadłowska et al. 2024a). Increased hypoxia in coastal bottom waters will also shift the redox zones upwards as oxygen and nitrate are no longer available as electron acceptors in the top sediments. Subsequently, labile OM will be buried in higher quantities below the SMTZ, providing more substrate for fermenters and ultimately methanogens. Furthermore, higher substrate availability leads to less competition and enables methanogens to thrive even in and above the SMTZ (Beulig et al. 2019, Coon et al. 2023).

In marine sediments, H_2_/CO_2_, and formate are the most prevalent methanogenic substrates (hydrogenotrophic methanogenesis), followed by acetate (acetoclastic methanogenesis; Liu and Whitman 2008). Methylated substrates, such as methanol and methylated amines, can also be converted to methane (methylotrophic methanogenesis), with or without H_2_ as a reductant, but their significance in marine environments has only gained more attention in recent years (Fischer et al. 2021, Tsola et al. 2024). As acetate and hydrogen can also be consumed efficiently by SRB, methylated compounds were long assumed to be “noncompetitive” substrates for methanogens. However, with the isolation of several methylotrophic SRB, this assumption should probably be revised (Sousa et al. 2018). Some methylated substrates such as trimethylamine and dimethylsulfide are abundant in vegetated and hypersaline sediments as breakdown products of algal osmolytes, and can be the main contributors to methane emissions (Kelley et al. 2014, Tsola et al. 2021). Also, many novel archaeal lineages, such as Korarchaeia and Thermoproteota, encode the genes for methylotrophic methanogenesis, and the first cultured representatives have recently been isolated (Kohtz et al. 2024, Krukenberg et al. 2024, Wu et al. 2024), expanding the occurrence and significance of this pathway.

Marine sediments generally harbor a diverse methanogenic community that can change rapidly with depth due to changing porewater chemistry and available substrates (Borrel et al. 2012, Zhang et al. 2020). Although many genes are shared in all pathways leading to methane production, most methanogens only encode the complete machinery for one methanogenic pathway, and members of the same order commonly have a very similar metabolism such as hydrogenotrophic Methanomicrobiales or Methanobacteriales (Liu and Whitman 2008). The order Methanosarcinales harbors the most diverse metabolisms including methylotrophic species such as the common marine sediment inhabitant *Methanolobus*, the metabolically versatile *Methanosarcina*, and the strictly acetoclastic *Methanosaeta* (*Methanothrix*). In addition, marine ANME archaea are also members of Methanosarcinales, but they belong to several different families (Chadwick et al. 2022).

Coastal systems often have lower salinity and less sulfate available for S-AOM. Thus, with the expanding methanogenic zone and increased methane production in eutrophic coastal sediments, the methanotrophic S-AOM community will be challenged with a methane surplus. As ANME archaea are notoriously slow-growing (Dale et al. 2008, Knittel et al. 2018, Lenstra et al. 2023), they might not be able to establish a stable methane-oxidizing zone (Egger et al. 2016). In the absence of sulfate, some ANME clades might be able to couple methane oxidation to metal oxides (metal-AOM) or nitrate AOM reduction (Wallenius et al. 2021), but the significance of these processes to the overall methane removal potential in coastal systems is not well quantified yet.

When methane production exceeds methanotrophy in coastal sediments, the methane cycle might become out of balance, resulting in increased benthic methane fluxes and emissions to the atmosphere (Venetz et al. 2023, Żygadłowska et al. 2023). With intensifying anthropogenic activity in coastal areas, eutrophication and hypoxia are predicted to further expand (Sinha et al. 2017, Breitburg et al. 2018). This may lead to even higher methane emissions from coastal sediments, making it urgent to understand the factors influencing the methane-cycling processes and microorganisms involved.

Here, we investigated the potential metabolic pathways for methane production and oxidation in anoxic sediments of eutrophic coastal marine Lake Grevelingen (The Netherlands). We aimed to answer the following questions: (1) What are the potential rates of methanogenesis and methanotrophy across the sediment? (2) What are the key microorganisms driving methane cycling in the different redox zones? (3) Which metabolic pathways are used for methane production and oxidation? These questions were addressed by a complementary set of experiments involving sediment incubation experiments to determine the rates of the various processes and DNA extractions together with 16S rRNA gene and metagenomic sequencing to assess the diversity and the potential methane-cycling processes.

## Materials and methods

### Study site and sampling

Lake Grevelingen, located in the southwestern part of the Netherlands, used to be an estuary until it was dammed, and partly re-connected to the North Sea in 1978 (Bannink et al. 1984), resulting presently in a salinity of 29–32 psu. The Scharendijke basin (45 m; 51.742°N; 3.849°E) is the deepest part of Lake Grevelingen, and the water column is subject to seasonal stratification, resulting in euxinic bottom waters in the summer months (Żygadłowska et al. 2024b). Sediment cores were collected in September 2020 during a sampling campaign on board R/V Navicula using a UWITEC gravity corer; two cores were retrieved for incubation experiments, one for DNA and porosity samples; one for porewater analysis and one for CH_4_ concentrations and isotopes. The cores for incubations were sliced anoxically, i.e. in a glovebag under a N_2_ atmosphere, at 5 cm intervals and stored anoxically, in gas-tight aluminum bags flushed with N_2_ gas, in the dark at 4°C until the start of incubation experiments within 2–4 months after sampling. The DNA samples were immediately frozen at −80°C after slicing of the core. Porewater samples for CH_4_, SO_4_^2−^, H_2_S, alkalinity, and δ^13^C–CH_4_ were collected directly after core retrieval. A detailed description of core retrieval and analysis for CH_4_ and porewater is described in Żygadłowska et al. (2023). While vertical seawater intrusion during coring can never be fully excluded (Jørgensen et al. 2024), we see little evidence for a significant impact on our porewater data for this site (Żygadłowska et al. 2024b, Klomp et al. 2025).

### Methanogenic incubations

Sediments from selected depths (5–15, 25–35, and 45–55 cm) were diluted with anoxic sulfate-free artificial seawater (ASW) medium in 1:1 ratio in an anoxic chamber and carefully homogenized. Then, 10 g of sediment slurry was transferred into 60 ml serum bottles, which were immediately stoppered with butyl rubber stoppers and capped. The ASW medium with a salinity of 32 ppt was composed of NaCl (26 g), MgCl_2_•6H_2_O (7.48 g), CaCl_2_•2H_2_O (1.45 g), KCl (0.6 g), and 20 ml of NaHCO_3_ solution (1 M) per liter of demineralized water. The pH was adjusted to 7.8. To remove any residual gases, the samples were flushed with argon (Ar), leaving 1.5 bar pressure, and the bottles were preincubated in the dark at 4°C for 3 weeks. Before substrate addition, the headspace was flushed again with Ar to remove any methane produced during preincubation and replaced with 99.5% Ar and 0.5% CO_2_. For each depth, two replicate samples were prepared to observe methane production without any added substrates (control). For three selected depths (5–15, 25–35, and 45–55 cm), the bottles were amended with acetate (5 mM), methanol (2 mM), methanol and H_2_ (2 mM; 8 mM), or CO_2_ and H_2_ (2 mM; 8 mM). Each condition was prepared in triplicate. The bottles were incubated at room temperature in the dark, while gently shaking. After 33 days of incubation, when all substrates appeared to be consumed as methane production rates returned to control levels, a second dose of acetate (10 mM), methanol (6 mM), methanol and H_2_ (24 mM), or CO_2_ (6 mM) and H_2_ (24 mM) was added to the bottles. This second dose was chosen to be two to three times larger than the initial dose in order to maintain methanogenic activity for a longer period. Methane and hydrogen concentrations in the headspace were measured with a HP5890 gas chromatograph (Agilent Technologies, Santa Clara, CA) equipped with a Porapak Q column and a thermal conductivity detector. Methane production rates were calculated for the first 3 days of incubation for all samples except for acetate, where substrate consumption only started after a 2-week lag phase and thus rates were calculated from days 17 to 24 when the methane increase was linear.

### Methanotrophic incubations

The methane oxidation potential was determined for five selected depth intervals (0–5, 5–10, 10–15, 15–20, and 25–35 cm). Ten grams of sediment were diluted in a 1:5 ratio with sulfate-free ASW in sterile 120 ml serum bottles, stoppered and capped. The headspace was replaced with 100% methane with 1.5 bar overpressure and the samples were preincubated for 3 weeks in the dark at 4°C to activate methane oxidizing microorganisms and deplete OM. To start the incubation, headspace was thoroughly flushed with Ar to remove all methane and amended with 20% ^13^C–CH_4_, 1% N_2_, and 1% CO_2_. The samples were amended with 5 mM Na_2_SO_4_ as electron acceptor for methane oxidation, or only ^13^C–CH_4_ as a control and incubated while gently shaking in the dark at room temperature. All conditions were prepared in duplicates. The ratio of ^13/12^C–CO_2_ was determined with a gas chromatography-mass spectrometer (GC–MS; Agilent 5975C inert MSD).

### DNA isolation

The *in situ* samples were defrosted and homogenized, and 0.2 g of the sediment slurry was bead-beaten on a TissueLyser LT (Qiagen, Venlo, The Netherlands) for 10 min at 50 Hz in a PowerBead tube from the DNeasy PowerSoil DNA isolation kit (Qiagen) before isolating the DNA according to manufacturer's instructions. The quantity and quality of isolated DNA eluted in sterile MilliQ water were assessed by NanoDrop 1000 (Thermo Fischer Scientific, Bremen, Germany), Qubit® 2.0 (Invitrogen, USA), and on 1% agarose gel. After isolation, DNA was immediately frozen at −20°C until further analysis.

### 16S rRNA gene amplicon sequencing and analysis

The amplification of the total archaeal 16S rRNA genes was performed with primers Arch349F (5′-GYGCASCAGKCGMGAAW-3′) and Arch806R (5′-GGACTACVSGGGTATCTAAT-3′ (Takai and Horikoshi 2000). The primers used for bacterial 16S rRNA gene amplification were Bac341F (5′-CCTACGGGNGGCWGCAG-3′ (Herlemann et al. 2011) and Bac806R (5′-GGACTACHVGGGTWTCTAAT-3′ (Caporaso et al. 2012). 16S rRNA gene amplicon sequencing was performed on the Illumina MiSeq Next Generation Sequencing platform by Macrogen (Seoul, South Korea) using Herculase II Fusion DNA Polymerase Nextera XT Index Kit V2, yielding 2 × 300 bp paired-end reads. The quality of raw reads was checked using FastQC (v0.11.5; (Andrew 2010). The paired-end reads were trimmed with Cutadapt (v1.18; Martin 2011) to remove adapters. The data were further processed using the DADA2 pipeline (v1.8; Callahan et al. 2016) in RStudio to cluster the reads into amplicon sequence variants, chimera removal and taxonomic classification using SILVA 16S rRNA gene database (v138.1; Quast et al. 2013). Microbial community data analysis was performed using the package phyloseq (v1.36.0; McMurdie and Holmes 2013) and visualized using ggplot2 (v3.3.5; Wickham 2016). The raw sequence reads can be accessed with BioProject accession PRJNA1167897.

### Metagenomic sequencing and gene-based analysis

Based on the results of 16S rRNA gene amplicon sequencing, five depths were chosen for further metagenomic sequencing: 0–2, 9–11, 15–17, 34–36, and 60–65 cm. DNA libraries were prepared using the TruSeq Nano DNA kit (550 bp) and paired-end sequencing with 2 × 151 bp sequence chemistry was performed on the Illumina NovaSeq 6000 platform by Macrogen, resulting in ~69 000 000 total reads per sample. FastQC v0.11.0 (Andrew 2010) was used to assess the quality of the reads. Trimming of adapters and low-quality data was performed using Bbduk (BBtools v37.76) using the built-in adapters.fa file, with a minimum quality of Q15 and a minimum read length of 100 bp. Error correction and coverage normalization were performed using Tadpole (mode = correct, *k* = 50) and BBnorm (target = 30, min = 2). Reads were assembled into contigs with metaSPAdes 3.15.5 (Nurk et al. 2017). Reads were mapped back to contigs with Bbmap (using slow = t for increased sensitivity). Four different algorithms were used for binning: MetaBAT 2.15 (Kang et al. 2019), MaxBin2 2.2.7 (Wu et al. 2016), CONCOCT 1.1.0 (Alneberg et al. 2014), and BinSanity 0.4.4 (Graham et al. 2017). High-scoring, dereplicated bins were generated using consensus binner DAS Tool 1.1.2 (Sieber et al. 2018). Bin completeness and contamination were assessed with CheckM 1.2.2 (Parks et al. 2015) and GTDB-tk 2.4.0 was used for taxonomic classification (Chaumeil et al. 2020).

Contigs from assemblies generated for each of the sequenced sediment layers were mined for genes involved in archaeal methane cycling. Amino acid translations of coding domain sequences were predicted using Prodigal 2.6.3 (Hyatt et al. 2010) and hmmsearch 3.4 (Eddy 1998) was used to identify target sequences based on hmmer profiles of corresponding KEGG numbers. Coverage of genes was estimated by using the coverage of their parent contigs. Contig coverages were calculated and normalized for transcript per million (TPM) using CoverM (Aroney et al. 2025). Protein sequences retrieved from the assembly by hmmer were then blasted against the SWISS-PROT database (Bairoch and Apweiler 2000) in order to eliminate spurious annotations. The raw sequence reads can be accessed with BioProject accession PRJNA1167897.

### Phylogenetic analysis of mcrA sequences

Previously published mcrA sequences were used as a reference (Chadwick et al. 2022), and the mcrA sequences were initially processed and aligned with MEGA12 (Kumar et al. 2024) and the maximum likelihood tree was generated with IQ-TREE 2.1.4-beta (Minh et al. 2020) using flags -m MFP-B 1000-nt AUTO. The tree was edited and annotated with iTol v7 (Letunic and Bork 2007). The aligned sequences can be accessed in Supplementary material.

## Results

### Porewater profiles and methanogenesis rates

**Figure 1.**
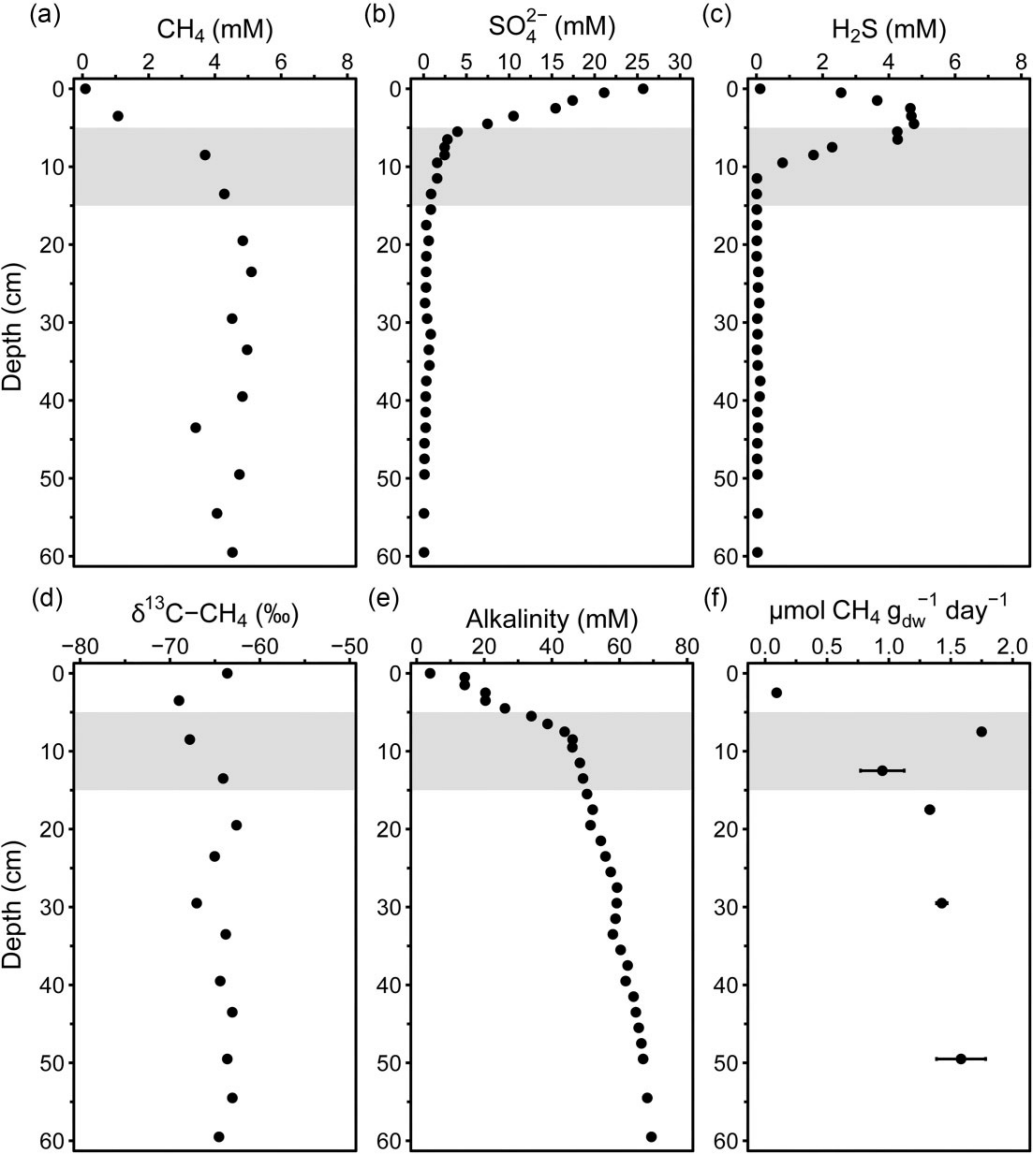
Porewater depth profiles of (a) methane, (b) sulfate, (c) sulfide, (d) δ^13^C-CH_4_, (e) alkalinity, and (f) potential methanogenesis rates without added substrates (*n* = 2). The gray shaded area indicates the SMTZ. Full porewater and solid phase profiles can be found in Żygadłowska et al. (2023).

The sediments in Scharendijke basin are characterized by a high sedimentation rate and OM input, which is reflected by the shallow SMTZ at ~5–15 cm below the sediment–water interface (Fig. 1a and b). In the top 5 cm, sulfate concentrations decrease rapidly from 25 to 3 mM as sulfate acts as the primary electron acceptor for OM degradation by SRB, which results in high sulfide concentrations in the porewater in this zone, peaking at 5 cm depth (up to 5 mM, Fig. 1c). The upward diffusing methane in the SMTZ was depleted in ^13^C, indicating that S-AOM probably does not contribute much to the isotopic signature (Fig. 1d), whereas methane was accumulating throughout the sediment as concentrations remained high (4–6 mM; Fig. 1a) down to 60 cm depth. The steep increase in alkalinity from top to 5 cm depth supports high OM degradation rates. The steady downward increase within the SMTZ suggests continued mineralization although at lower rates, which could be advantageous to methanogens competing with SRB for substrates (Fig. 1e). Methane production was observed at all incubated depths from 0–5 to 45–55 cm, albeit at a slow rate (0.1 ± 0.02 µmol CH_4_ g_dw_^−1^ day^−1^) in the top 5 cm (Fig. 1f). Also, the highest methanogenesis rate (1.7 ± 0.01 µmol CH_4_ g_dw_^−1^ day^−1^) was measured in the SMTZ at depths between 5 and 10 cm where the sulfate concentration was still above 2 mM, which is unexpected for marine sediments. Below the SMTZ, the potential methane production rate remained stable at about 1.2 µmol CH_4_ g_dw_^−1^ day^−1^.

### High methanogenesis potential across sediment and substrates

**Figure 2.**
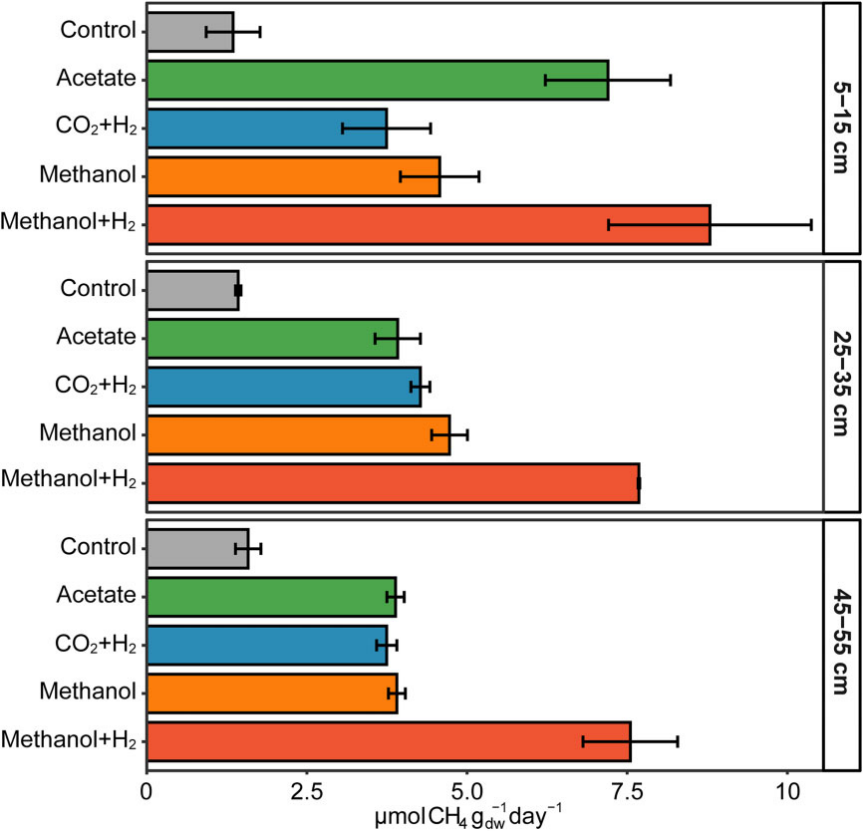
Potential methane production rates from different methanogenic substrates in Lake Grevelingen sediment from three different sediment depth intervals. Error bars represent variation between duplicates (control) or triplicates.

At all depths, all added methanogenic substrates initiated methane production well above the control levels (Fig. 2). From all the substrates, the highest potential methanogenesis rates were measured in the 5–15 cm incubations with methanol and H_2_ as substrates, with a maximum of 8.8 µmol CH_4_ g_dw_^−1^ day^−1^. Acetoclastic, hydrogenotrophic, and methylotrophic (without H_2_) methanogenesis rates were similar (∼3.7–4.7 µmol CH_4_ g_dw_^−1^ day^−1^) across the various sediment intervals, except for the acetate amended bottles from the 5–15 cm layer where methane production was fastest (7.2 µmol CH_4_ g_dw_^−1^ day^−1^) after the initial lag phase (Fig. S1a). Here, CO_2_/H_2_ amended sediment had the slowest methane production rate, suggesting that other methanogenic pathways may be more active in the SMTZ. Also, at the deepest depth (45–55 cm) the measured methane production rates were comparable to the shallower depths, indicating that there is still a substantial methanogenic community at this depth that can be easily activated. Overall, all depths showed a high potential for methanogenesis from all the main methanogenic substrates. When the incubations were provided with a second dose of substrate, the adapted methanogenic community showed an even higher maximum methane production rate of 30 µmol CH_4_ g_dw_^−1^ day^−1^ with methanol/H_2_ (Fig. S1a).

### Methane oxidation detected in narrow zone below SMTZ

**Figure 3.**
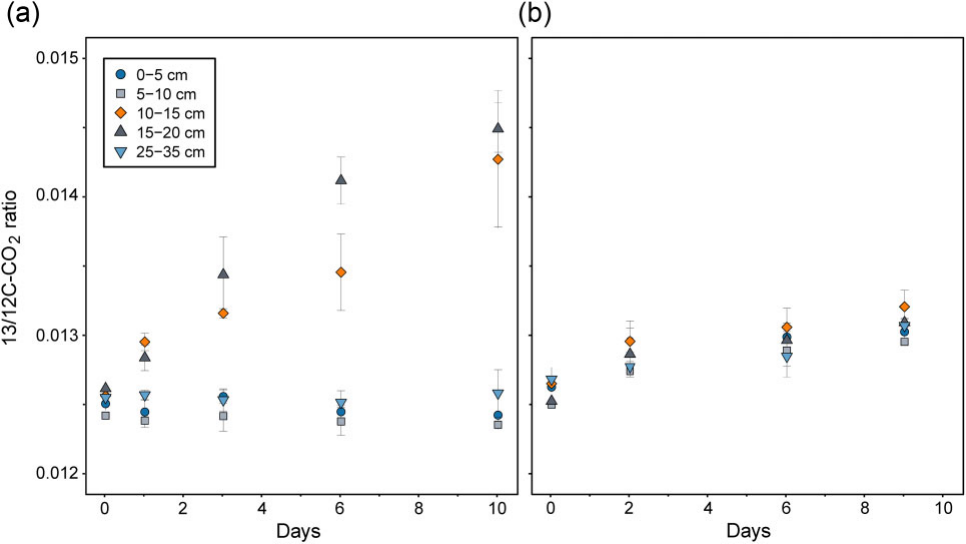
Potential S-AOM activity for the five sediment depth intervals. The increase in the ratio of ^13/12^C–CO_2_ in the headspace gas phase in batch incubations amended with (a) ^13^C–CH_4_ and sulfate or (b) ^13^C–CH_4_ only.

In addition to the methane production potential, we investigated the change in anaerobic methane oxidation potential with depth. The increase in the ratio of ^13/12^C–CO_2_ is the result of oxidation of ^13^C–CH_4_ and is therefore taken as an indication for S-AOM. For the five tested depths above, at and below the SMTZ, active methane oxidation was only observed at 10–15 and 15–20 cm depth (Fig. 3a), with the highest increase in ^13^C–CO_2_ at 15–20 cm, directly below the SMTZ (Fig. 1a). The ^13/12^C ratio started to increase after 1 day, suggesting an active S-AOM community in this narrow zone around the SMTZ. However, the methane oxidation rate was slow, an average of 0.0019 ± 0.001 µmol CH_4_ g_dw_^−1^ day^−1^ (Fig. S2), which is two to three orders of magnitude lower than the average potential methanogenesis rate (Fig. 1). In the control samples without added electron acceptors, the ^13/12^C ratio increased only slightly in all depths, suggesting some residual electron acceptor and AOM activity at all depths, albeit at much lower levels than in the sulfate-amended samples (Fig. 3b).

### Methanogens and ANME inhabit specific niches in the sediment

**Figure 4.**
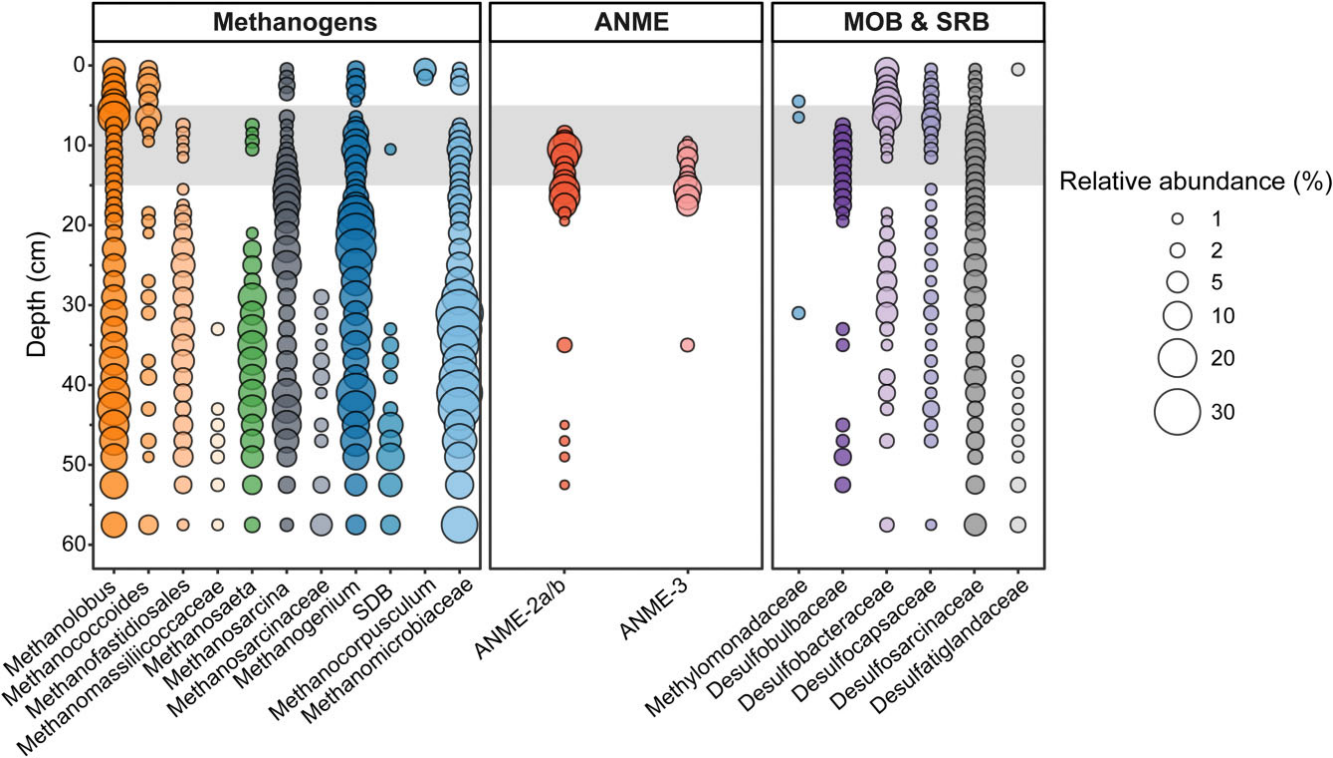
The relative abundance of methane-cycling archaea from total archaeal reads and methanotrophic and SRB from total bacterial reads. The gray box indicates the SMTZ at 5-15 cm depth. Methanogens are listed and grouped by the potential metabolic pathway in different colors; methylotrophic (orange; n = 4); acetoclastic (green; n = 1); generalist/flexible (gra; n = 2); hydrogenotrophic (blue; n = 3). MOB, methane-oxidizing bacteria (blue; n = 1). Only taxa with >1% relative abundance in at least two depths are included. SDB, putative genus of Methanomicrobiaceae as classified by SILVA

The high-resolution 16S rRNA gene amplicon sequencing revealed a diverse and abundant methanogenic community across the top 60 cm of sediment, with distinct variations across redox zones (Fig. 4). Methylotrophic *Methanolobus* and *Methanococcoides* were the most abundant methanogens in the top 10 cm, reaching up to 20% relative abundance of archaeal community, suggesting that methane produced in the top layer originates primarily from methylated substrates. However, hydrogenotrophic methanogens from the *Methanomicrobiaceae* family (*Methanogenium* and unclassified) were also prevalent in the top layers with maximum 13% relative abundance at 9 cm depth. The SMTZ did not harbor an overall high abundance of methanogens, but methylotrophic taxa were more abundant in the top sulfide-rich zone, whereas *Methanosaeta* and *Methanogenium* were more abundant around 10 cm (2% and 10%, respectively), the shallowest depth where sulfide was no longer detected. Below the SMTZ, methanogen relative abundance increased reaching 90% of archaeal reads at 40 cm, with hydrogenotrophic methanogens dominating. Strictly acetoclastic *Methanosaeta* were commonly found below 30 cm with 5%–10% abundance, and increased when *Methanosarcina* and *Methanogenium* declined, indicating differences in OM composition and substrate availability.

In contrast, ANME archaea were mainly detected in and below the narrow SMTZ, aligning with observed S-AOM activity (Fig. 3a). ANME-2a/b accounted for up to 15% of the archaeal community at 10–11 cm, while ANME-3 reached 10% at 15–16 cm. Each clade appeared to have a specific niche at the SMTZ, with ANME-2a/b dominating at higher sulfate concentrations, and ANME-3 when sulfate was below 0.1 mM. The distribution of ANME correlated with *Desulfobulbaceae* reads in the SMTZ, a putative bacterial partner in S-AOM, which was mainly found between 8 and 19 cm, while other SRB were more broadly distributed. On genus level, the SEEP–SRB1 group was more abundant in the SMTZ than at other depths, suggesting a relationship with ANME (Fig. S2). Below 35 cm, ANME were detected at <2% abundance, likely representing buried cells. Despite high methanogen abundance, high methane production potential and high sulfate concentrations in the top 5 cm, no aerobic or anaerobic methanotrophs were detected. Bacterial *Methylomonadaceae* reads were detected around 5 cm depth, where they likely represent buried cells from the water column MOB community (Venetz et al. 2023).

**Figure 5.**
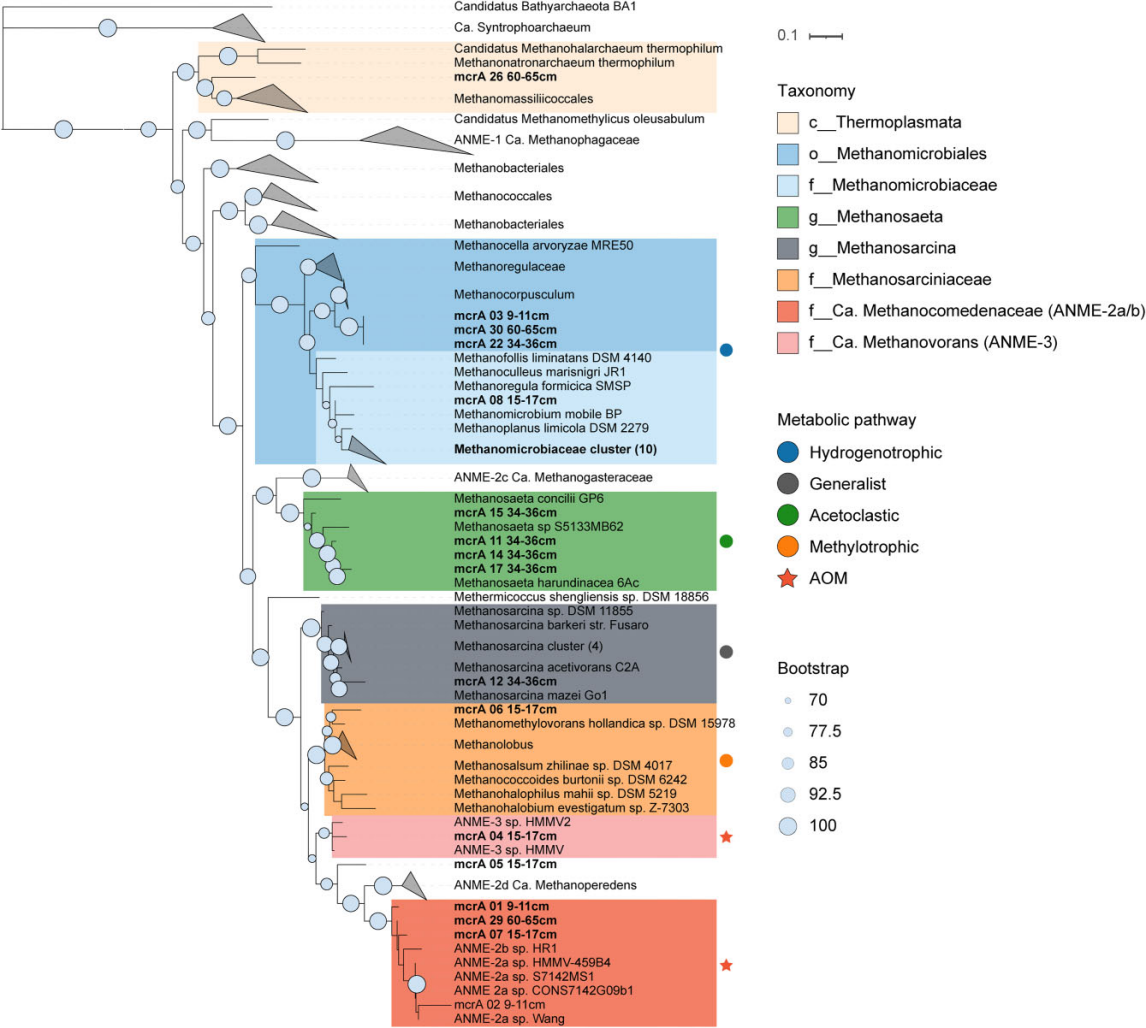
Maximum likelihood phylogenetic tree of mcrA (partial) sequences. The sequences from this study are in bold, with the recovery depth indicated in the name. The clades with detected sequences are colored, the symbols represent the most probable metabolic methane pathway. Bootstrap support values above 70% for 1000 replicates are shown in the beginning of the node*. Candidatus* Bathyarchaeia BA1 was used as the outgroup.

**Figure 6.**
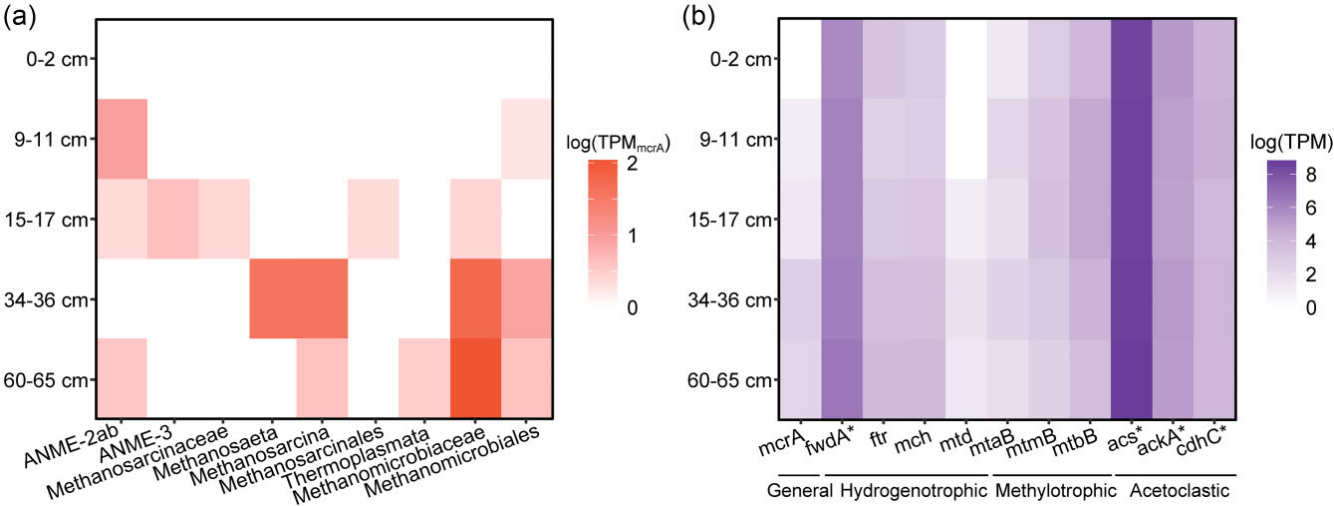
The abundance of methane-cycling genes in the metagenomes at different sediment depths. The taxonomy and abundance of mcrA (partial) genes recovered from the metagenomes (a) and the distribution of marker genes involved in different methanogenesis pathways (b). mcrA, methyl-coenzyme M reductase alpha subunit; fwdA, formylmethanofuran dehydrogenase subunit A; ftr, formylmethanofuran-tetrahydromethanopterin N-formyltransferase; mch, methenyltetrahydromethanopterin cyclohydrolase; mtd, methylenetetrahydromethanopterin dehydrogenase; mtaB, methanol-5-hydroxybenzimidazolylcobamide Co-methyltransferase; mtmB, methylamine-corrinoid protein co-methyltransferase; mtbB, monomethylamine methyltransferase; acs, acetyl–CoA synthase; ackA, acetate kinase; cdhC, acetyl–CoA decarbonylase/synthase complex beta subunit. Asterisks indicate nonmethanogen specific genes.

No ANME or methanogen metagenome-assembled genomes could be recovered from the metagenomes from five different depths, so we focused on gene-based analysis of methane-cycling genes. We recovered 31 (partial) *mcrA* sequences and their phylogenomic analysis identified both ANME and methanogenic genes (Fig. 5), in line with the 16S rRNA gene. amplicon results. Four *mcrA* sequences clustered with *Ca*. Methanocomedenaceae family (ANME-2a/b), and one with *Ca*. Methanovorans (ANME-3; Fig. 5). The majority of the *mcrA* sequences found in the SMTZ were identified as ANME; ANME-2a/b was found both at 9–11 cm and 15–17 cm, whereas ANME-3 *mcrA* sequences were only present at 15–17 cm (Fig. 6a). The rest of the sequences clustered with known methanogenic taxa; majority (*n* = 14) with hydrogenotrophic *Methanomicrobiaceae*. The most *mcrA* sequences were detected at 34–36 cm, and they indicated potential for all main pathways as the reads from this depth clustered with *Methanosaeta* (*n* = 4), *Methanosarcina* (*n* = 4), and *Methanomicrobiaceae* (*n* = 5). One *mcrA* sequence clustered with methylotrophic members of *Methanosarcinaceae* (15–17 cm), and one with potentially H_2_-dependent methylotrophic Thermoplasmata members (60–65 cm).

As *mcrA* sequences were not recovered from all depths, we quantified the abundance of marker genes for all main methanogenic pathways. Consistent with amplicon sequencing results, marker genes for all major methanogenic pathways were detected (Fig. 6b). Hydrogenotrophic genes (*fwdA, ftr, mch*) were abundant throughout the sediment, whereas *mtd* was detected only below 15 cm. For methylotrophic methanogens, marine sediments can provide several different substrates, such as methanol and methylamines, and all pathways require different methyltransferases (Kurth et al. 2020). Of these, *mtbB*, marker gene for dimethylamine dependent methanogenesis was highly abundant in all depths, followed closely by *mtmB* (monomethylamine-dependent). Interestingly, both genes, as well as *mtaB* (methanol-dependent) had the highest number of reads in the SMTZ. However, due to the high similarity of methyltransferase genes/proteins, the assignment to specific genes was not always successful such as for mttb (trimethylamine dependent). For acetoclastic methanogenesis, the marker genes for acetate activation are also found in several bacteria and other archaea, and thus both *acs* and *ackA* that are used by *Methanosaeta* and *Methanosarcina*, respectively, were highly abundant throughout the sediment.

## Discussion

We studied the impacts of anthropogenic eutrophication on the microbial methane cycle in the sediments of Scharendijke basin (Lake Grevelingen), a potential hotspot for atmospheric methane emissions due to a very high sedimentation rate, OM input, and seasonally anoxic bottom waters linked to water column stratification (Żygadłowska et al. 2024b). Żygadłowska et al. (2023) reported a high *in situ* methane production rate of 2 µmol cm^−2^ d^−1^ (0.075 µmol g^−1^ d^−1^ in the upper meter of the sediment) based on a reactive transport model, while finding little geochemical evidence for methane oxidation. In addition, Żygadłowska et al. (2024b) showed high benthic release of methane from the sediment in all seasons. To better understand what causes the imbalance in the microbial methane cycle and to discover which key microbes are involved, we combined sediment profiles, rate and activity measurements, as well as 16S rRNA gene profiling and metagenomics.

### High methane production in Scharendijke basin

After summer stratification, the SMTZ was narrow and close to the sediment–water interface (5–15 cm depth) as sulfate, the main electron acceptor for OM degradation, is rapidly depleted due to prolonged bottom water anoxia, resulting in a high sulfide peak and a sharp increase in alkalinity in the top 5 cm. High sedimentation rates can lead to shoaling of the SMTZ (LaRowe et al. 2020), and in Scharendijke basin the sedimentation rate has been reported to be up to 20 cm year^−1^ (Klomp et al. 2025), resulting in a high flux of labile OM and a sharp redox zonation. Thus, there are plenty of substrates for methanogenesis, which is reflected in the high *ex situ* potential methanogenesis rates (maximum 1.7 µmol CH_4_ g_dw_^−1^ day^−1^_;_ average 1.2 µmol CH_4_ g_dw_^−1^ day^−1^) across the top 60 cm of Scharendijke sediment, which are high for a coastal marine environment. However, we note that the average rate is ∼16-fold higher than the *in situ* methanogenesis rate of 0.075 µmol g^−1^ d^−1^ calculated by Zygadlowska et al. (2023). This is probably due to a higher incubation temperature, in sulfate-free ASW, preincubation to suppress other microbial processes, and the lack of incubation data from below 60 cm where the methanogenesis rates are expected to decrease significantly due to lower supply of labile OM. Unexpectedly, the highest potential methane production rate was measured at 7 cm depth, within the SMTZ, and methanogenesis was detected even in the top 2 cm of the sediment in the sulfate reduction zone. In deeper marine sediments, methanogenesis is often limited to the zone below the SMTZ where sulfate is depleted and methanogens are no longer outcompeted by SRB for their shared substrates such as acetate and CO_2_/H_2_ (Liu and Whitman 2008). In such a setting, most, if not all, methane is thus produced below the ANME methane filter and consequently removed in the SMTZ. However, in coastal sediments with high OM input, such as the Baltic Sea (Maltby et al. 2018, Dalcin Martins et al. 2024) and the Peruvian margin (Maltby et al. 2016), active methanogenesis was detected in the sulfate-reducing zone. Incubation studies showed methylotrophic methanogenesis as dominant in this zone, but 16S rRNA gene amplicon results revealed a presence of metabolically diverse communities. Thus, our results support the previous findings and strengthen the argument that methanogens are not outcompeted by SRB in coastal sediments and that the two microbial guilds can co-exist, even close to the sediment–water interface.

### Imbalance between methanogenesis and AOM potential

The methanogenesis incubations suggested that Scharendijke sediments harbor a high potential for methane production via all major methanogenic substrates and pathways, both within and below the SMTZ. In contrast, despite high methane availability, S-AOM potential was only detected in a narrow zone in or just below the SMTZ (10–20 cm), indicating constriction of methane oxidation to a small zone. Thus, methane can be produced at high rates below, within, and even above the methane oxidation zone, creating an obvious imbalance in the methane cycle.

Based on our potential rate measurements, it is difficult to determine a dominant methane production pathway in Scharendijke sediments. The rates per substrate varied in different sediment depth intervals, suggesting differences in both substrate availability and methanogenic community composition. However, hydrogenotrophic methanogenesis rates were the lowest measured in two out of three depths. At all depths, methanol and H_2_ addition resulted in the highest methanogenesis rates, but co-occurring methane production solely through methanol and H_2_ consumption by hydrogenotrophic methanogens, driven by CO_2_ release from fermentation, cannot be excluded. Apart from methanol+H_2_, acetoclastic methanogenesis was the fastest in the SMTZ, followed by methanol. All acetate incubations had a lag phase, which might indicate that *Methanosarcina* was the active methanogen in these samples as they have been shown to require some time to adjust upon acetate addition if they were used to a different substrate *in situ* (Zinder and Elias 1985, de Jong et al. 2018). At 25–35 cm, methanol initiated a higher rate, and even in the deepest layer, methanol-dependent methanogenesis was as fast as in the samples with acetate and CO_2_/H_2_. Therefore, our results suggest that methylotrophic methanogenesis can play an important role in Scharendijke basin sediments and could even be the main methane source at certain depths. Similarly, both methylotrophic and hydrogenotrophic methanogenesis were found to contribute to methane production in sulfate-rich zones of coastal Mediterranean Sea sediments (Zhuang et al. 2018) and in Aarhus Bay sediments (Xiao et al. 2018). Thus, high OM input driven by eutrophication may support a diverse methanogenic population across the sediment.

### Large metabolic and phylogenetic diversity of methanogens across the sediment

In line with the activity assays, both 16S rRNA gene amplicon sequencing and metagenomic analysis revealed a highly diverse methanogenic community across the sediment profile with potential for all the major pathways. Common marine methylotrophs *Methanolobus* and *Methanococcoides* were dominant in the sulfate-reduction zone. Also Tsola et al. (2021) observed both genera enriched in dimethylsulfide-dependent methanogenic incubations, indicating that they can use a wide array of methylated compounds for methane production. Furthermore, the strictly H_2_-dependent methylotrophic methanogens Methanofastidiosales and Methanomassiliicoccales were present, but mainly below the SMTZ. Other methylated substrates such as monomethylamine, dimethylamine, and trimethylamine are common in marine sediments (Zhuang et al. 2017), and a different methyltransferase is needed for each substrate (Kurth et al. 2020). In addition to *mtaB* (used in methanol metabolism), we found both *mtmB* (monomethylamine) and *mtbB* (dimethylamine) reads in high abundances at every depth, indicating that methylotrophic methanogenesis may indeed play a large role in methane production in Scharendijke basin. Hydrogenotrophic marker genes were also ubiquitous and correlated well with 16S rRNA gene amplicon results. However, these genes are also used in other methanogenic pathways (Kurth et al. 2020), making it hard to pinpoint the distribution of hydrogenotrophic methanogens alone. Similarly, acetoclastic methanogenesis shares most acetate-activation genes with multiple acetogenic bacteria, as well as microbes utilizing the Wood–Ljungdahl pathway for carbon fixation, a significant source of carbon in anoxic marine sediments (Sansone and Martens 1982, Kremp and Müller 2021). Therefore, the wide distribution and high abundance of *acs, ackA*, and *cdhC* across the sediment profile is likely due to the presence of multiple acetate-utilizing guilds, in addition to methanogens. Thus, we can conclude that acetate is an important intermediate in the carbon cycle across the sediment profile of Lake Grevelingen.

The phylogenomic analysis of the *mcrA* sequences also supported a high diversity as well as some niche separation of different methanogenic clades. The *mcrA* sequences belonged to two different phyla; Halobacterota and Thermoplasmatota. Halobacterota comprises most canonical hydrogenotrophic and methylotrophic methanogens, as well as the acetoclastic *Methanosaeta* and generalist *Methanosarcina*. The *mcrA* abundance and diversity were highest at 34–36 cm, suggesting that even at this depth there still is enough labile OM to support several methanogenic pathways. In the deepest examined layer, however, the hydrogenotrophic methanogens were clearly the most abundant, which is common for marine sediments (Bojanova et al. 2023).

The most ANME *mcrA* reads clustered within the ANME-2a/b family named *Candidatus* Methanocomedenaceae, common habitant of cold methane seeps (Chadwick et al. 2022). The ANME-3 *mcrA* sequence clustered closely to ANME-3, or *Ca*. Methanovorans sequences from a deep-sea submarine mud volcano. This indicates that coastal sediments harbor many typically deep-sea ANME clades. As for the syntrophic bacterial partners, our data suggests that *Desulfobulbaceae* (DBB) are the bacterial partners for S-AOM in the SMTZ as they correlate with ANME abundance. Interestingly, this DBB branch of ANME–SRB syntrophic partners is usually associated exclusively with ANME-3 (Lösekann et al. 2007). ANME-2a/b is commonly detected in aggregates with the SEEP–SRB1 clade, part of the *Desulfosarcinaceae* family (Schreiber et al. 2010, Murali et al. 2023). Indeed, SEEP–SRB1 reads comprised more than half of the total *Desulforsarcinaceae* reads in the SMTZ, thus suggesting that they also had a role in S-AOM, potentially with ANME-2a/b.

### The SMTZ as a poor methane filter inhabited by ANME archaea and SRB

S-AOM is the major pathway for methane removal in marine sediments, and is potentially the main AOM pathway in Scharendijke basin sediments (Bhattarai et al. 2017, Cassarini et al. 2019). Previously, ANME-3 was identified as the most abundant ANME, with *Desulfobulbus* SRB as their potential syntrophic partner. However, our high-resolution 16S rRNA gene profile combined with metagenomics sequencing identifies ANME-2a/b as the key player in AOM over ANME-3. Curiously, the two clades seemed to inhabit separate niches within the SMTZ, with ANME-2a/b reads and *mcrA* sequences being most abundant in the top layers of the SMTZ at 9–11 cm, whereas ANME-3 was mainly detected at 15–17 cm, where sulfate was almost depleted. ANME-2a/b is commonly found in other coastal sediments, as well as in sulfate-rich top layers of methane seeps (Chen et al. 2022, Deng et al. 2025). ANME-3 are usually associated with sites with fluid flow, such as mud volcanoes and cold seeps (Niemann et al. 2006, Vigneron et al. 2013). Even though ANME-2a/b reads were present at 5–10 cm depth in both of our gene-based analyses, no AOM activity was observed in this zone. It is unclear what, and if something, is inhibiting AOM activity in the upper layers of the SMTZ. In coastal sediments, sulfide toxicity has been previously been shown to inhibit ANME activity in brackish coastal sediments (Dalcin Martins et al. 2024). Alternatively, Lapham et al. (2024) suggested that high OM would lead to surplus H_2_, which would inhibit AOM thermodynamically in Baltic Sea sediments. Moreover, ANME archaea are notoriously slow-growing with doubling times varying between ∼2 and 7 months, even in laboratory conditions (Nauhaus et al. 2007, Meulepas et al. 2009), with *in situ* doubling times estimated to be up to ∼130 days based on a gene-based reactive transport model (Lenstra et al. 2023). Thus, the lack of appreciable S-AOM could also be associated with the short residence time in the zone where sulfate is still available as was suggested by Egger et al. (2016). With a high sedimentation rate of up to 20 cm per year, ANME would not be able to establish a stable methane filter within the narrow SMTZ before getting buried below the sulfate zone. We hypothesize that both biomass-limitation, due to slow growth, and high sulfide concentrations due to high OM input, could be important factors to explain the inefficient methane filter in Scharendijke basin sediments and in similar organic-rich coastal settings, such as the brackish lagoon Cape Lookout Bight (Coon et al. 2023) and the Baltic Sea (Lapham et al. 2024). However, follow-up studies are required to better understand which environmental factors affect the ANME methane filter in coastal sediments. Comparison of different coastal sites with similar characteristics are needed to map the broader scale of the imbalanced methane cycle in eutrophic sediments. As seasonal stratification may get more common and prolonged in many coastal systems, seasonal monitoring of changes in the methane cycle in systems prone to bottom water deoxygenation is crucial to understand how the methane cycle dynamics will develop in the future.

Finally, we also looked at the presence of aerobic MOB. Gammaproteobacterial *Methylomonadaceae* reads were detected around the SMTZ but were likely not active in the sediment. Although MOB have several survival strategies under oxygen limitation (Reis et al. 2024, Schorn et al. 2024), at the time of sampling, water column δ^13^CH_4_ profiles and potential methane removal rates did not indicate MOB-mediated methane removal in the anoxic bottom water (Venetz et al. 2023, Zygadlowska et al. 2023). Thus, the detected *Methylomonadaceae* probably represent buried cells from the water column MOB community, and a significant contribution of MOB to methane removal in the anoxic sediment therefore seems unlikely.

The methane oxidation activity, together with the 16S rRNA gene amplicon profile, shows that the methane oxidation potential is low in the sediments of the Scharendijke basin, and that methanotrophs are vastly outnumbered by methanogens, explaining the high methane emissions to the water and atmosphere observed at this site.

## Conclusion

We investigated the microbial methane cycling potential in the anoxic sediments of the coastal Scharendijke basin (Lake Grevelingen). We conclude that the microbial methane cycle in the sediment is favored toward methanogenesis, making this system a source of atmospheric methane. The top 65 cm of the sediment harbors a highly diverse archaeal methanogen community, which is potentially distributed along specific niches based on substrate availability. We also find that methylotrophic methanogens are abundant, highlighting their key role in methane production in coastal sediments. The metabolic diversity is reflected in the high methanogenic rates from all major methanogenic pathways, even in and above the SMTZ. In contrast, methane removal is slow and restricted to the narrow SMTZ, and only in this zone ANME archaea are present, with separate niches for ANME-2a/b and ANME-3. Aerobic methanotrophy does not seem to play a role. Together our results indicate that anthropogenic eutrophication of coastal waters potentially increases methane emissions from these systems, as the changes favor methanogenesis over methanotrophy. Future research is needed to study the seasonal dynamics in these systems to understand the balance between the different methanogenic pathways and the methane biofilter in coastal sediments and how that influences methane emissions.

## Supplementary Material

fiaf075_Supplemental_File
