# Supplementary material for: A ubiquitous and diverse methanogenic community drives microbial methane cycling in eutrophic coastal sediments
Source: FEMS Microbiol Ecol. 2025 Jul 12;101(8):fiaf075. doi: 10.1093/femsec/fiaf075 (PMC12287602; doi:10.1093/femsec/fiaf075)
Supplement: fiaf075_Supplemental_File [file fiaf075_supplemental_file.docx]

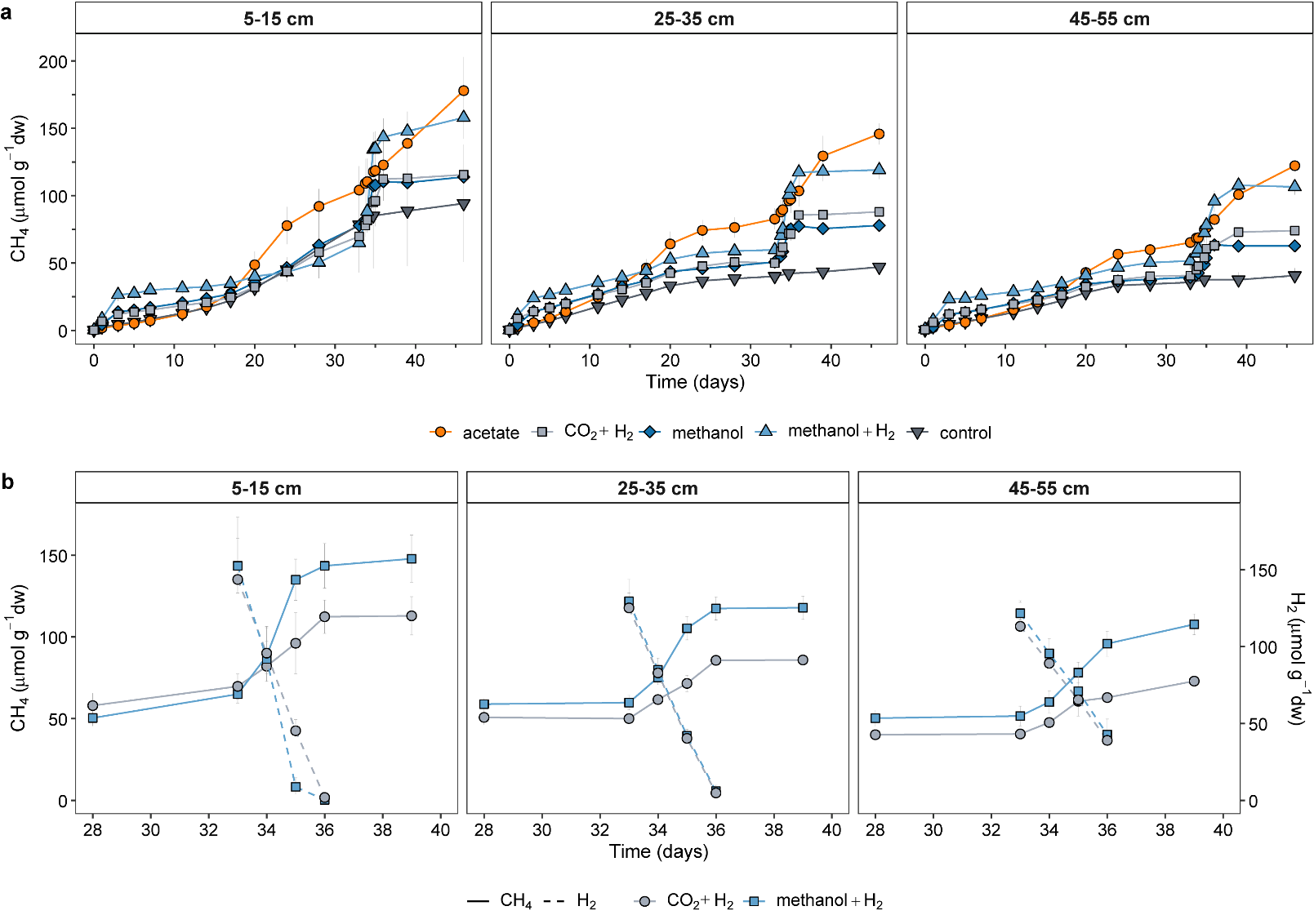
**Supplementary figures**

**Fig. S1.** Total methane production in substrate-amended and control samples over the whole experiment (a) and during the second substrate addition, where H_2_ consumption was also measured (b). Substrates were added on days 0 and 33.

**
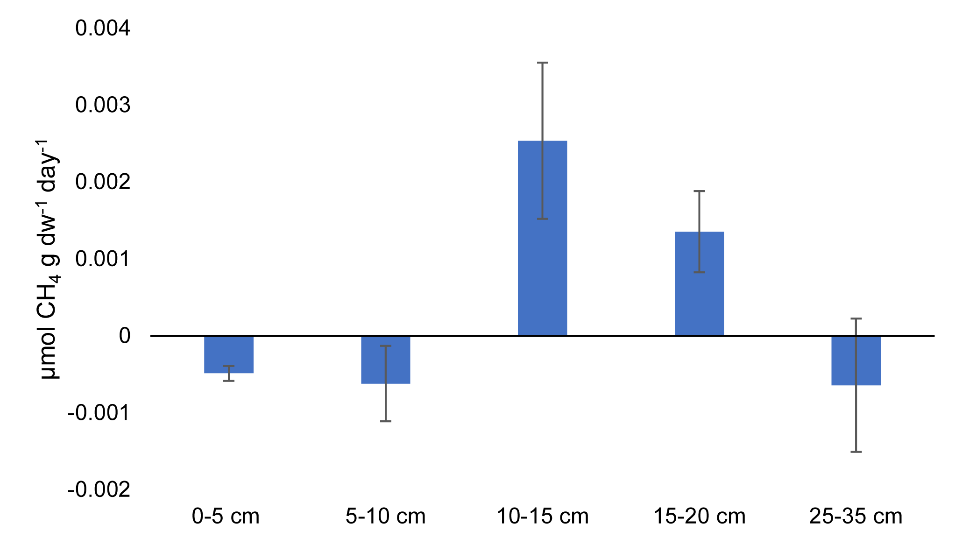
**

**Fig. S2.** The S-AOM rate at different sediment depths in incubations amended with sulfate and ^13^C-CH_4_, calculated from the increase in ^13^C-CO_2_ molar ratio during the first 6 days of the incubations.

**
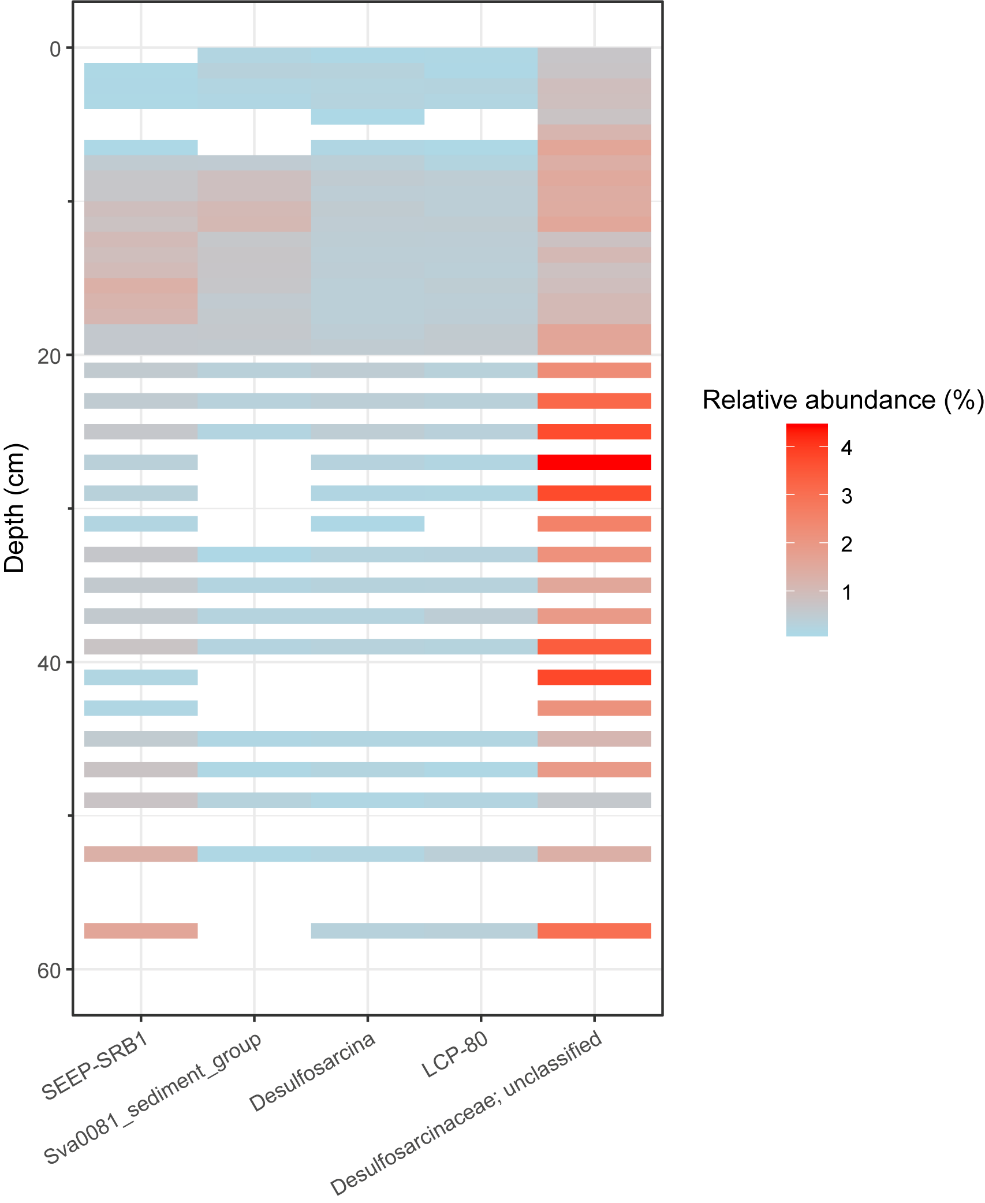
**

**Fig. S3.** Relative abundance of *Desulfosarcinaceae* 16S rRNA gene reads at genus level.
